# Psychometric Properties of the Dutch Cognitive Avoidance Questionnaire

**DOI:** 10.5334/pb.522

**Published:** 2020-07-02

**Authors:** Elien Vanderveren, Elise Debeer, Miet Craeynest, Dirk Hermans, Filip Raes

**Affiliations:** 1 Center for the Psychology of Learning and Experimental Psychopathology, Faculty of Psychology and Educational Sciences, University of Leuven, BE; 2 Department of Applied Psychology, Thomas More University of Applied Sciences, BE; 3 Department of Applied Psychology, Howest University of Applied Sciences, BE

**Keywords:** cognitive avoidance, Cognitive Avoidance Questionnaire, psychometric evaluation, brooding

## Abstract

Various psychological disorders, such as major depressive disorder and anxiety disorders, share cognitive avoidance as one of the main mechanisms underlying symptom severity and maintenance. A valid and reliable measure that covers a broad array of cognitive avoidance strategies is therefore instrumental, though currently greedily missing. The Questionnaire d’Évitement Cognitif (QEC) was developed as a comprehensive instrument measuring five cognitive avoidance strategies. The current study aimed to evaluate the psychometric properties of the Dutch Cognitive Avoidance Questionnaire (CAQ-NL) in three samples (*N* sample 1 = 607; *N* sample 2 = 357; *N* sample 3 = 448). Confirmatory factor analysis supported the hypothesized five-factor structure of the CAQ-NL. Reliability analysis showed good to excellent internal consistency for the CAQ-NL and its five subscales. Furthermore, Multi-Group CFA revealed that the CAQ-NL demonstrated configural and metric invariance across the three samples. Convergent validity of the CAQ-NL was supported by substantial correlations with brooding, with more cognitive avoidance being related to more brooding. In addition, cognitive avoidance was negatively associated with psychological well-being and positively with symptoms of depression and anxiety, which corroborates the instrument’s concurrent validity. Moreover, the CAQ-NL was predictive of depressive symptoms six months later, supporting its predictive validity. In sum, results of the present study provide support for the validity and reliability of the CAQ-NL.

Within the study of the development and treatment of anxiety disorders, great emphasis has been put on the role of cognitive avoidance. The latter covers a broad array of behaviors, such as suppressing unwanted thoughts or images, distracting oneself or replacing aversive thoughts with more pleasant alternatives (Barlow, 2002). Engaging in such behaviors has been related to the development and maintenance of various anxiety disorders (Spinhoven et al., 2017), like obsessive-compulsive disorder (Purdon, 2004; Reuman et al., 2018), posttraumatic stress disorder (Shipherd & Beck, 2005), and generalized anxiety disorder (Newman et al., 2013; Spinhoven et al., 2014). Within the framework of generalized anxiety disorder (GAD), pervasive worry holds a prominent role (Aikins & Craske, 2001; Borkovec et al., 2004). More specifically, worrying about minor issues is considered an attempt to avoid being exposed to topics perceived as potentially threatening or harmful (Borkovec et al., 2004). In support of this, cognitive-behavioral therapy targeting pathological worry in patients suffering from GAD has shown to be effective in reducing anxiety symptoms (Dugas & Ladouceur, 2000; Hanrahan et al., 2013).

Besides being a fundamental element of several anxiety disorders, cognitive avoidance, in addition to other vulnerability factors, plays a role in the development and maintenance of major depressive disorder. Ferster (1973) stated that depression is often characterized by different cognitive avoidant behaviors such as rumination, putting of making decisions or avoidant thinking about goals or solutions. Empirical evidence corroborates this theoretical perspective on depression. More specifically, the use of cognitive avoidant strategies relates to the concurrent experience of depressive symptoms (Ottenbreit & Dobson, 2004; Quigley et al., 2017). It is also predictive of the development and course of depression over time (Blalock & Joiner, 2000; Dickson et al., 2012; Spinhoven et al., 2014). One example of a cognitive avoidant strategy that has been established as a cognitive vulnerability factor for the onset and course of depression is rumination (Nolen-Hoeksema et al., 2008; Raes, 2005; Raes et al., 2003). Brooding and reflection are commonly considered to be the two components of rumination. According to Treynor and colleagues (2003), brooding is the passive pondering on one’s negative mood, whereas reflection refers to intentionally focusing on one’s negative mood with the goal of problem solving. Brooding has been established as the maladaptive component of rumination (Griffith & Raes, 2015). By focusing attention on the causes and potential consequences of negative feelings, one avoids engaging in active problem solving (Moulds et al., 2007). This hypothesis is supported by numerous studies reporting a positive association between rumination and measures of cognitive avoidance (Ottenbreit et al., 2014; Raes et al., 2009; Spinhoven et al., 2017), in particular with the brooding component of rumination (Moulds et al., 2007; Quigley et al., 2017).

Given the central role of cognitive avoidance in both the conceptualization and treatment of several psychological disorders, a valid and reliable measure that encompasses a broad array of cognitive avoidance strategies is instrumental. Though there are a number of valid and reliable measures of cognitive avoidance available, they often focus on one aspect of cognitive avoidance or they measure avoidance in correspondence to one specific life event. The *Questionnaire d’Évitement Cognitif* (QEC; Gosselin et al., 2002) addresses this need for a more encompassing measure. The QEC measures five cognitive avoidance strategies: thought substitution, transformation of images into thoughts, distraction, avoidance of threatening stimuli, and thought suppression. As such, the QEC is a comprehensive measure that could prove a valuable tool in both research and clinical practice. The validity and reliability of the QEC and its English translation (Cognitive Avoidance Questionnaire or CAQ; Sexton & Dugas, 2008) have been evaluated in prior studies. Though these studies support the instrument’s validity and reliability, they provide no information about its ability to predict future symptomatology or its invariance across samples.

The purpose of the present study was to evaluate the psychometric properties of the Dutch Cognitive Avoidance Questionnaire (CAQ-NL). The study had two main objectives: (1) To examine the replicability of the hypothesized five-factor structure; and (2) to evaluate its reliability, convergent, concurrent, and predictive validity. Convergent validity was evaluated by examining the association between the CAQ-NL and brooding. We hypothesized that, in line with the literature, higher scores on the CAQ-NL would relate to more brooding. Concurrent validity was evaluated by examining how scores on the CAQ-NL relate to outcome measures such as symptoms of anxiety, depression, and psychological well-being. We predicted that higher scores on the CAQ-NL would be related to more symptoms of depression and anxiety and to less psychological well-being. Finally, the CAQ-NL was examined as a predictor of depressive symptoms at a later time point in order to evaluate its predictive validity.

## Method

### Participants

Three samples were collected between 2009 and 2019. The first sample was a community sample which consisted of 625 adults. Among these, 18 participants had omissions for one or more CAQ-NL items (two participants had omissions for all CAQ-NL items, two for 15 items, one for seven and one for two items, and, finally, 12 participants had omissions for one item). Little’s MCAR test indicated the data were missing completely at random (*df* = 291; *χ*^2^ = 297.84; *p* > .05). These 18 participants were therefore excluded for the analyses, resulting in a sample of 607 participants. Participants of the final sample were between 17 and 84 years old (*M* = 33.53; *SD* = 13.68), of whom 207 were men (34.1%) and 400 were women (65.9%). A total of 45.6% of participants had completed post-secondary higher education (college or university). The remaining 54.4% indicated their highest achieved educational level to be that of high school or lower. A majority of 56.6% of participants reported to be currently working, 33.3% were students, and 10% were unemployed (due to retirement, disability or other). A follow-up took place 6 months later, at which time depressive symptoms were re-assessed. Of the original 607 participants, a group of 526 completed the follow-up (86.66%).

The second sample consisted of 357 first-year psychology students, of whom 51 were men (14.3%) and 306 were women (85.7%). Participants’ age ranged between 17 and 26 years old (*M* = 18.29; *SD* = .97).

The third sample was a mixed student population consisting of 448 students attending college or university. Their ages ranged from 17 to 47 years (*M* = 21.65; *SD* = 3.01); 64 of them were men (14.3%) and 384 were women (85.7%). Of these students, a group of 40.8% were enrolled in the faculty of psychology and educational sciences, 17.2% studied science, 8.9% followed courses in the medical field, 6.3% studied law, 5.6% studied business or economics, 6.5% were following courses in social sciences, and 14.7% were enrolled in other courses (e.g., languages, teacher training etc.). As the data for both Sample 2 and 3 were collected through Qualtrics, which offers the option to not allow items to remain unanswered, there were no missing data for both these samples.

## Procedure

### Translation

The instructions and items of the original QEC were translated to Dutch, after which back-translation to French was performed by the Leuven Language Institute, an institute with great expertise in translating research articles and questionnaires. These back-translations were then reviewed by Dr. Patrick Gosselin, one of the authors of the QEC. Some minor adjustments were performed after the review by Dr. Gosselin, leading to the final Dutch version of the QEC.

### Data Collection

Sample 1 was a convenience sample of community-dwelling adults that were recruited by master students using their personal social networks and extensions. Participants filled out various self-report questionnaires, among which measures of cognitive avoidance, brooding, and symptoms of depression. Depression symptoms were re-assessed six months later.

For the second and third sample, data were collected through the online survey platform Qualtrics. Sample 2 exclusively consisted of first-year psychology students. In order to gain course credit, students could participate in an online survey that consisted of various self-report questionnaires, among which measures of cognitive avoidance, brooding, symptoms of depression and anxiety, and psychological well-being. Participants for the third sample were recruited through flyers on various social media platforms. The survey consisted of measures of cognitive avoidance and brooding. Students could win a gift voucher to the value of €50 by participating.

Prior to taking part in these studies, participants signed an informed consent. Study 1 was approved by the Ethical Committee of the Faculty of Psychology and Educational Sciences of KU Leuven. Both study 2 and 3 were approved by the Social and Societal Ethics Committee of KU Leuven.

## Measures

### Cognitive Avoidance

The *Questionnaire d’Évitement Cognitif* (QEC; Gosselin et al., 2002) is a 25-item self-report questionnaire designed to measure a broad array of cognitive avoidance strategies frequently used when faced with intrusive thoughts. The QEC comprises five subscales that reflect five different cognitive avoidance strategies: Thought Substitution, Transformation of Images into Thoughts, Distraction, Avoidance of Threatening Stimuli, and Thought Suppression. Each subscale is measured by five items. Participants rate the applicability of each item on a five-point scale, ranging from 1 (*not at all applicable*) to 5 (*very applicable*), with higher scores indicating more cognitive avoidance.

The QEC has previously been established as a reliable and valid measure of cognitive avoidance. The total scale as well as the five subscales individually show great internal consistency (α = .95 for all items and α ranges between .71 and .90 for the five subscales) and test-retest reliability (*r* = .81 over an interval of four weeks). In addition, it has been found to correlate moderately strong with worry and worry-related processes (on average *r* ranging between .40 and .50) (Gosselin et al., 2002). CFA on the five-factor structure of the CAQ, the English translation of the QEC (Sexton & Dugas, 2008), revealed acceptable, though at the lower boundaries of adequacy, model fit (CFI = .88; RMSEA = .08; SRMR = .06). Moreover, the CAQ demonstrated good to excellent internal consistency (α = .95 for all items and α ranges between .73 and .89 for the five subscales) and test-retest reliability (*r* = .85 over an interval between four to six weeks).

### Brooding

Brooding was assessed using the brooding subscale of the *Ruminative Response Scale* (Raes et al., 2009; Treynor et al., 2003), which contains five items. Cronbach’s alpha of the brooding scale was .76 in Sample 1, .75 in Sample 2, and .76 in Sample 3.

### Depression and Anxiety

Symptoms of depression and anxiety were assessed using the depression and anxiety subscales of the *Depression Anxiety Stress Scale-21* (DASS-21; de Beurs et al., 2001; Lovibond & Lovibond, 1995). The validity and reliability of the DASS-21 have been demonstrated in numerous studies (e.g., Osman et al., 2012; Page et al., 2007). The internal consistency of the depression scale was .88 in Sample 1 and .92 in Sample 2. The anxiety scale had an internal consistency of .84 in Sample 2.

### Psychological Well-Being

The *Flourishing Scale* consists of eight items that measure the respondents self-perceived overall psychological well-being (Diener et al., 2010). Items describe various domains of functioning, such as feelings of competence, experiencing positive relationships with others etc. Cronbach’s alpha of the Flourishing Scale in Sample 2 was .91.

## Data-Analysis

AMOS 25.0 was used to perform confirmatory factor analysis (CFA). The theorized five-factor model as well as a more parsimonious one-factor model were tested. A Maximum Likelihood estimator was used. Following standard conventions, model fit was assessed by the following four criteria: (1) a non-significant Chi-square test; (2) the Comparative Fit Index (CFI) with values between .90 and .95 indicating acceptable fit and values above .95 indicating good model fit; (3) the Root Mean Square Error of Approximation (RMSEA) with a value below .08 indicating acceptable fit and below .06 indicating good model fit; and (4) the Standardized Root Mean Square Residual (SRMR) with a value below .08 indicating good model fit (Hu & Bentler, 1999). Given the large sample sizes present in this study, a significant Chi-square test is not necessarily an indicator of bad fit as this index is sensitive to sample size. Therefore, more weight was assigned to the other three fit indices in evaluating overall model fit.

Multi-Group CFA was performed to assess configural and metric invariance across samples, for which the following four fit indices were used: (1) the delta (Δ) Chi-Square; (2) the ΔCFI for which values should not exceed .01; (3) ΔRMSEA for which values should be below .015; and (4) ΔSRMR for which values should be below .030 (Chen, 2007). As we noted earlier, the CFI, RMSEA and SRMR are less sensitive to large sample sizes, therefore invariance was evaluated by determining change in these three fit indices rather than solely relying on the Chi-square test (Cheung & Rensvold, 2002).

Cronbach’s alphas were computed in all three samples to assess the reliability of the CAQ-NL and its five scales. Interpretation of internal consistency was as follows: <.60 = insufficient; .60 to .69 = marginal; .70 to .79 = acceptable; .80 to .89 = good; and .90 or higher = excellent (Barker et al., 1994). Inter-correlations between CAQ-NL scales and correlations between CAQ-NL scales and measures of brooding, well-being, and symptoms of depression and anxiety were assessed by computing Pearson correlation coefficients. It was assessed whether these correlations could withstand a Bonferroni correction for multiple testing. Finally, linear regression analyses were performed to investigate cognitive avoidance as a predictor of depressive symptoms over time. All these additional analyses were performed using SPSS 25.0.

## Results

### Confirmatory Factor Analysis

The hypothesized five-factor model was first tested on Sample 1. Model fit indices showed the goodness-of-fit of the model to be borderline acceptable compared to conventional standards, though still adequate (*df* = 265; *χ*^2^ = 1290.76; *p* < .05; CFI = .87; RMSEA = .079; SRMR = .056). CFA on Samples 2 (*df* = 265; *χ*^2^ = 803.63; *p* < .05; CFI = .90; RMSEA = .076; SRMR = .06) and 3 (*df* = 265; *χ*^2^ = 994.62; *p* < .05; CFI = .88; RMSEA = .078; SRMR = .06) yielded similar results. For these three samples, the RMSEA and SRMR indicated acceptable to good model fit. The CFI values were marginally acceptable. Taken together, the goodness-of-fit of the hypothesized five-factor model was not great, yet still adequate and very similar to the fit of the English version (i.e., CFI = .88 and RMSEA = .08; SRMR = .06; Sexton & Dugas, 2008). Factor loadings of all CAQ-NL-items for each sample are presented in Table 1.

**Table 1:** Factor Loadings Based on CFA of the CAQ-NL in Samples 1, 2, and 3.

Item		Sample		

		1	2	3

**Thought Substitution**				

4.	I think about things that concern me as if they were occurring to someone else.	.58	.65	.41
11.	I think about trivial details so as not to think about important subjects that worry me.	.70	.75	.66
17.	I think about past events so as not to think about future events that make me feel insecure.	.58	.69	.53
20.	I think about many little things so as not to think about more important matters.	.72	.69	.71
25.	I think about things that are worrying other people rather than thinking about my own worries.	.45	.52	.40
**Transformation of Images into Thoughts**				

3.	I replace threatening mental images with things I say to myself in my mind.	.60	.69	.62
15.	I keep saying to myself in my head to avoid visualizing scenarios (a series of mental images) that frighten me.	.71	.76	.76
19.	When I have mental images that are upsetting, I say things to myself in my head to replace the images.	.64	.68	.62
23.	Rather than having images of upsetting events form in my mind, I try to describe the events using an internal monologue (things that I say to myself in my head).	.69	.71	.63
24.	I push away the mental images related to a threatening situation by trying to describe the situation using an internal monologue.	.70	.72	.64
**Distraction**				

8.	I distract myself to avoid thinking about certain disturbing subjects.	.75	.80	.77
10.	I often do things to distract myself from my thoughts.	.75	.77	.79
12.	Sometimes I throw myself into an activity so as not to think about certain things.	.66	.72	.69
13.	To avoid thinking about subjects that upset me, I force myself to think about something else.	.79	.76	.73
21.	Sometimes I keep myself occupied just to prevent thoughts from popping up in my head.	.64	.72	.73
**Avoidance of Threatening Stimuli**				

7.	I sometimes avoid objects that can trigger upsetting thoughts.	.68	.69	.64
9.	I avoid people who make me think about things that I do not want to think about.	.74	.74	.73
16.	Sometimes I avoid places that make me think about things I would prefer not to think about.	.81	.74	.80
18.	I avoid actions that remind me of things I do not want to think about.	.83	.78	.79
22.	I avoid situations that involve people who make me think about unpleasant things.	.77	.76	.76
**Thought Suppression**				

1.	There are things that I would rather not think about.	.67	.77	.81
2.	I avoid certain situations that lead me to pay attention to things I do not want to think about.	.71	.73	.76
5.	I have thoughts that I try to avoid.	.78	.84	.83
6.	I try not to think about the most upsetting aspects of some situations so as not to be too afraid.	.67	.69	.67
14.	There are things I try not to think about.	.78	.82	.86

*Note*: All factor loadings are significant at *p* < .001.

Regarding the more parsimonious one-factor model, results indicated that the five-factor model shows a more adequate fit in all three samples and is therefore preferable. The fit-indices for the one-factor model were as follows: *df* = 275, *χ*^2^ = 2014,85, *p* < .05, CFI = .78, RMSEA = .10, SRMR = .06 (Sample 1); *df* = 275, *χ*^2^ = 1222.46, *p* < .05, CFI = .82, RMSEA = .10, SRMR = .07 (Sample 2); and *df* = 275, *χ*^2^ = 1727.73, *p* < .05, CFI = .75, RMSEA = .11, SRMR = .07 (Sample 3).

Results of the Multi-Group CFA indicated that configural invariance was obtained as both the RMSEA and SRMR were indicative of good model fit and the CFI was marginally acceptable (*df* = 795, *χ*^2^ = 3092.04, *p* < .05; CFI = .88; RMSEA = .05; SRMR = .06). Although the Chi-square test was significant, we can cautiously conclude that metric invariance was obtained as well, as the change in the other three fit indices was well below conventional cut-off scores (*df* = 40, Δ*χ*^2^ = 57.74, *p* = .03; ΔCFI = .00; ΔRMSEA = .00; ΔSRMR = .00). This indicates that the number of factors, the overall pattern of factor loadings, and the individual factor loadings were consistent across the three samples.

### Reliability

The internal consistency of the CAQ-NL and its five subscales, as evaluated by Cronbach’s alpha, ranged from good to excellent. Cronbach’s alpha of Thought Substitution resided on the lower bound of adequacy, especially in Sample 3, though was still acceptable. Overall, internal consistency scores were highly consistent with the French and English questionnaire (see Method section). Table 2 contains an overview of Cronbach’s alpha scores of each CAQ-NL scale in the three samples separately. Inter-correlations between the five CAQ-NL scales are presented in Table 3.

**Table 2:** Internal Consistency, Mean, and Standard Deviation of the Five Subscales of the CAQ-NL in Samples 1, 2, and 3.

	Sample 1			Sample 2			Sample 3		

	α	*M*	*SD*	α	*M*	*SD*	α	*M*	*SD*

Substitution	.73	1.64	.63	.79	2.18	.83	.66	1.91	.67
Transformation	.79	1.69	.71	.82	2.25	.91	.79	1.97	.80
Distraction	.84	2.02	.81	.87	2.68	.99	.86	2.55	.95
Avoidance	.87	1.74	.84	.85	2.41	.98	.86	2.12	.93
Suppression	.85	2.38	.90	.88	3.00	.95	.89	2.72	.94
CAQ_Total	.94	1.89	.66	.95	2.50	.81	.94	2.25	.71

*Note*: One-way ANOVA’s showed significant differences between the samples on all CAQ-NL dimensions. Post-hoc comparisons revealed that mean avoidance scores in Sample 2 were consistently higher compared to the other two samples, while scores were consistently lowest in Sample 1.

**Table 3:** Inter-Correlations Between the Five Subscales of the CAQ-NL in Samples 1, 2, and 3.

	Sample	Transformation	Distraction	Avoidance	Suppression	CAQ_Total

Substitution	1	.64	.66	.66	.59	.82
	2	.75	.67	.67	.58	.84
	3	.63	.60	.50	.54	.77
Transformation	1		.66	.59	.59	.81
	2		.71	.70	.69	.88
	3		.62	.54	.60	.81
Distraction	1			.67	.69	.87
	2			.69	.77	.89
	3			.63	.70	.87
Avoidance	1				.66	.85
	2				.69	.87
	3				.66	.82
Suppression	1					.85
	2					.86
	3					.86

*Note*: All correlations are significant at *p* < .001 level and withstand a Bonferroni correction for multiple testing.

### Convergent Validity

Pearson correlation coefficients between the CAQ-NL and brooding are presented in Table 4. The CAQ-NL total score and subscales showed moderate positive associations with brooding in all three samples.

**Table 4:** Pearson Correlations Between the Five Subscales of the CAQ-NL and Measures of Brooding (Samples 1, 2, and 3), Depression (Samples 1 and 2), and Anxiety and Psychological Well-Being (Sample 2).

	Sample	Brooding	Depression	Anxiety	Well-Being

Substitution	1	.41	.34		
	2	.44	.48	.53	–.28
	3	.46			
Transformation	1	.35	.27		
	2	.42	.44	.49	–.21
	3	.46			
Distraction	1	.40	.31		
	2	.44	.41	.47	–.22
	3	.49			
Avoidance	1	.39	.33		
	2	.45	.49	.54	–.30
	3	.48			
Suppression	1	.45	.36		
	2	.50	.40	.44	–.20
	3	.56			
CAQ_Total	1	.48	.38		
	2	.52	.51	.57	–.28
	3	.60			

*Note*: All correlations are significant at *p* < .001 level and withstand a Bonferroni correction for multiple testing. No data regarding the association between the CAQ-NL and measures of anxiety and well-being were gathered in Samples 1 and 3.

### Concurrent Validity

As shown in Table 4, CAQ-NL total score and subscales were found to positively correlate with self-reported symptoms of anxiety and depression. In addition, significant negative associations between CAQ-NL subscales and psychological well-being were observed.

### Predictive Validity

The CAQ-NL total score was predictive of depressive symptoms 6 months later, *β* = .15, *t*(523) = 4.65, *p* < .001, even while controlling for concurrent depressive symptoms. Regarding the five subscales of the CAQ-NL, only Thought Substitution and Thought Suppression were predictive of future depressive symptoms over and above the other cognitive avoidance strategies (see Table 5).

**Table 5:** Summary of Linear Regression Analysis With the Five Subscales of the CAQ-NL and Depressive Symptoms at T1 as Predictors and Depressive Symptoms at T2 (6 Months Later) as Criterion.

	*β*	*SE*	*t*	*η*_p_^2^

Depression_T1	.27	.04	6.97***	.08
Substitution	.10	.05	2.13*	.01
Transformation	–.03	.04	–.75	.00
Distraction	–.04	.04	–1.04	.00
Avoidance	.04	.04	1.06	.00
Suppression	.09	.03	2.57*	.01

*Note: N* = 526. * *p* < .05; *** *p* < .001.

### Discussion

The present study aimed to evaluate the psychometric properties of the Dutch translation of the Cognitive Avoidance Questionnaire. The study had two main objectives: (1) To examine the replicability of the hypothesized five-factor structure; and (2) to evaluate its reliability, convergent, concurrent, and predictive validity. With regards to the first objective, results from CFA lend support for the hypothesized five-factor model in all three samples, thereby replicating the structure of the original QEC (Gosselin et al., 2002). This five-factor model was preferable over a more conservative one-factor model. Although the overall goodness-of-fit was less than what conventional standards usually recommend, the fit was acceptable and, more importantly, very similar to the fit of the original version.

The CAQ-NL’s invariance across three separate samples was investigated as well. Multi-group CFA revealed that the CAQ-NL demonstrated configural and metric invariance, meaning that the underlying pattern of factors as well as factor loadings are consistent across different samples. In other words, the subscales of the CAQ-NL and their respective items are interpreted similarly and have the same psychological meaning across various groups.

For the second objective, the CAQ-NL’s reliability, convergent, concurrent, and predictive validity were evaluated. Concerning the instrument’s reliability, both the CAQ-NL total scale and its five subscales demonstrated good to excellent internal consistency. The subscale Thought Substitution resided on the lower bound of adequacy, yet still within the range of acceptability.

In line with the theoretical perspective on rumination as a cognitive avoidance strategy (Moulds et al., 2007), the CAQ-NL showed significant positive associations with the maladaptive brooding component of rumination. This lends support for the convergent validity of this cognitive avoidance measure. Higher scores on the CAQ-NL were, in addition, related to more symptoms of depression and anxiety and were associated with lower psychological well-being, which confirms the scale’s concurrent validity. These significant associations with depressive and anxiety-related symptoms are in correspondence with earlier research findings (e.g., Newman et al., 2013; Quigley et al., 2017).

The CAQ-NL was not only concurrently associated with depressive symptoms, it contributed significantly to the prediction of future depressive symptoms over and above baseline depressive symptoms. More specifically, Thought Substitution and Thought Suppression were predictive of depressive symptoms over and above the other cognitive avoidant strategies and baseline depressive symptoms. This lends support for the CAQ-NL’s predictive validity.

As cognitive avoidance is a central mechanism that underlies symptom severity and maintenance in depression and anxiety disorders, the CAQ-NL, which measures a broad array of cognitive avoidant strategies in a valid and reliable way, is a clinically valuable instrument. For example, in evaluating the clinical effectiveness of therapeutic interventions, clinicians can, in addition to outcome measurements, conduct the CAQ-NL as a process measure. By doing so, it can be determined if the intervention has an effect on cognitive avoidance as the underlying process as well as on the symptomatology. As this study has demonstrated the CAQ-NL’s ability in predicting future depressive symptomatology, assessing cognitive avoidance over the course of therapy could provide an indication of the course of the depressive symptoms.

Replication of the factorial structure and validity of the CAQ-NL in three large and diverse samples are definite strengths of the present study. There are, however, some noteworthy limitations that should be taken into account. First, the evaluation of convergent validity was limited to examining the relation between the CAQ-NL and brooding. A more extensive examination of the association between the CAQ-NL and other cognitive avoidance questionnaires, such as the Cognitive-Behavioral Avoidance Scale (CBAS; Ottenbreit & Dobson, 2004), White Bear Suppression Inventory (WBSI; Wegner & Zanakos, 1994), the Penn State Worry Questionnaire (PSWQ; Meyer et al., 1990), or the Action and Avoidance Questionnaire (AAQ-II; Bond et al., 2011) could provide additional support for its convergent validity. Second, the CAQ-NL’s discriminant validity was not evaluated. This would be a valuable addition in determining its psychometric quality. Third, test-retest reliability of the CAQ-NL could not be evaluated in the present study. Future research could therefore contribute to the findings of this study by examining the stability of scores on the CAQ-NL over time. Finally, the current study exclusively evaluated the CAQ-NL’s validity and reliability in community samples. A replication in clinical samples is therefore required.

In conclusion, the current study provides support for the five-factor structure and the internal consistency of the Dutch Cognitive Avoidance Questionnaire. Multi-group comparisons reveal that the CAQ-NL’s subscales and their respective items are interpreted similarly across various groups. The study contributes to the renewed and growing interest in the role of cognitive avoidance in depression by demonstrating the predictive power of cognitive avoidance, more specifically of Thought Substitution and Thought Suppression, in predicting depressive symptoms over time. In addition, the observed significant associations with brooding, symptoms of anxiety and depression, and psychological well-being confirm the CAQ-NL’s convergent and concurrent validity. In sum, results of the present study provide support for the validity and reliability of the CAQ-NL.
